# Dose Reduction Strategies for Pregnant Women in Emergency Settings

**DOI:** 10.3390/jcm12051847

**Published:** 2023-02-25

**Authors:** Carmine Picone, Roberta Fusco, Michele Tonerini, Salvatore Claudio Fanni, Emanuele Neri, Maria Chiara Brunese, Roberta Grassi, Ginevra Danti, Antonella Petrillo, Mariano Scaglione, Nicoletta Gandolfo, Andrea Giovagnoni, Antonio Barile, Vittorio Miele, Claudio Granata, Vincenza Granata

**Affiliations:** 1 Division of Radiology, “Instituto Nazionale Tumori IRCCS Fondazione Pascale—IRCCS di Napoli”, 80131 Naples, Italy; 2 Medical Oncology Division, Igea SpA, 80013 Naples, Italy; 3 Department of Emergency Radiology, University Hospital of Pisa, 56124 Pisa, Italy; 4 Department of Translational Research, Academic Radiology, University of Pisa, 56124 Pisa, Italy; 5 Diagnostic Imaging Section, Department of Medical and Surgical Sciences & Neurosciences, University of Molise, 86100 Campobasso, Italy; 6 Division of Radiology, “Università degli Studi della Campania Luigi Vanvitelli”, 81100 Naples, Italy; 7 Division of Radiology, Azienda Ospedaliera Universitaria Careggi, 50134 Florence, Italy; 8 Italian Society of Medical and Interventional Radiology (SIRM), SIRM Foundation, Via della Signora 2, 20122 Milan, Italy; 9 Department of Clinical and Experimental Medicine, University of Sassari, 07100 Sassari, Italy; 10 Diagnostic Imaging Department, Villa Scassi Hospital-ASL 3, Corso Scassi 1, 16121 Genoa, Italy; 11 Department of Clinical, Special and Dental Sciences, University Politecnica Delle Marche, Via Conca 71, 60126 Ancona, Italy; 12 Department of Radiology, University Hospital “Azienda Ospedaliera Universitaria delle Marche”, Via Conca 71, 60126 Ancona, Italy; 13 Department of Radiology, Careggi University Hospital, Largo Brambilla 3, 50134 Florence, Italy; 14 Department of Radiology, G. Gaslini Institute, IRCCS, 16147 Genova, Italy

**Keywords:** optimization, dose reduction, emergency, pregnancy, imaging

## Abstract

In modern clinical practice, there is an increasing dependence on imaging techniques in several settings, and especially during emergencies. Consequently, there has been an increase in the frequency of imaging examinations and thus also an increased risk of radiation exposure. In this context, a critical phase is a woman’s pregnancy management that requires a proper diagnostic assessment to reduce radiation risk to the fetus and mother. The risk is greatest during the first phases of pregnancy at the time of organogenesis. Therefore, the principles of radiation protection should guide the multidisciplinary team. Although diagnostic tools that do not employ ionizing radiation, such as ultrasound (US) and magnetic resonance imaging (MRI) should be preferred, in several settings as polytrauma, computed tomography (CT) nonetheless remains the examination to perform, beyond the fetus risk. In addition, protocol optimization, using dose-limiting protocols and avoiding multiple acquisitions, is a critical point that makes it possible to reduce risks. The purpose of this review is to provide a critical evaluation of emergency conditions, e.g., abdominal pain and trauma, considering the different diagnostic tools that should be used as study protocols in order to control the dose to the pregnant woman and fetus.

## 1. Introduction

In modern clinical practice, there is an increasing dependence on imaging techniques in most settings, and especially in emergency settings. Consequently, there has been an increase in the frequency of imaging examinations and with it an increased risk of radiation exposure. In this context, a critical setting is a pregnant woman who requires proper diagnostic management during an emergency. Provision of the right care to a pregnant woman could cause anxiety, which could result in delayed, inappropriate, or underutilization or overutilization of imaging [1].

Ionizing radiation fetal exposure is one of the main concerns for care practitioners, although the risk of radiation effects on fetus is correlated to radiation dose and radiation target as does the stage of gestation [2]. With regard to the target, radiation examinations on the expecting mother’s head, neck, and extremities cause a very low fetal irradiation. However, diagnostic fluoroscopy and abdomen and pelvis computed tomography (CT) studies provide a more substantial dose.

Regarding the gestation stage, it is believed that, during the first 2 weeks after fertilization, radiation is responsible for an “all or nothing” event, which could determine either normal development of the fetus, without effects, or a spontaneous abortion. The dose threshold for inducing a spontaneous abortion is estimated to be 50 to 100 mGy or higher, which corresponds to a greater dose than a single CT study should ever reach [2,3,4,5]. However, the rate of this event is about 50% when a fetus is subjected to a radiation-based examination during the early phases of gestation [3]. After the 2nd week, the main risk is the teratogenesis, which is common between the 2nd and 25th weeks, especially between the 8th and 15th weeks. Teratogenesis is due to both rapid organ development and differentiation at this stage, at a dose higher than 50 to 100 mGy [2,4]. There are different kinds of damage that can occur regardless of the dose, the so-called radiation stochastic effects, among which carcinogenesis is the most significant [6,7,8,9,10,11,12,13,14,15,16,17,18,19,20,21,22,23,24,25]. Anyway, the risk that carcinogenesis will occur is low if diagnostic examinations are managed appropriately. Although doubts exist, this risk is similar in the second and third trimesters and part of the first trimester. The lifetime attributable risk of developing cancer is approximately 0.4% per 10 mGy fetal dose [3]. So, the total fetal radiation exposure through pregnancy should be known if a pregnant woman is subjected to multiple necessary examinations. In this regard, the mother should be clearly informed of the possibility of this event, and it should be part of the informed consent [6]. A radiation level lower than 50 mGy is not correlated to fetal injury or abortion [8]; so, considering that dose reduction and optimization are the main goals in the radiological field, in accordance with the “as low as reasonably achievable” (ALARA) principle, the fetus should not be exposed to doses approaching this threshold in most of the imaging examinations used in clinical practice [9,10,11,12,26].

## 2. Communicating Benefits and Risks

Pregnant women exposed to radiation often request counselling about the associated radiation exposure and fetal risks [25]. In this context, it is critical that the counsellor be well-versed in the potential adverse effects correlated to the different levels of radiation exposure, so as not to unintentionally increase apprehension. Inappropriately, some care givers have inadequate knowledge of the ionizing radiation biological effects and of the possibility of using alternative non-ionizing diagnostic techniques. Consequently, inadequate counselling may cause more harm than the radiation exposure itself [27,28,29]. The conditions surrounding inadvertent radiation exposure of a pregnant woman or any patient due to inaccurate counselling on the radiation risk may have medicolegal implications [12,15,23,26,30].

Since many of the complex issues dealt with in this paper are not clearly known by non-healthcare professionals, nor among all healthcare professionals, a valuable information source for women could be their obstetric care provider. However, the obstetrician usually does not have the knowledge to offer proper counselling for all exposure conditions, and other experienced professionals, such as radiologists, should be proposed [25,31,32,33,34,35,36,37,38,39,40,41,42,43,44,45,46,47,48,49]. In addition, it would be appropriate that the consultation include not only verbal communication, but a written report with supporting information and clarifications as well [10,11,12,13,14,15,50,51,52,53,54,55,56,57,58,59,60,61,62,63,64,65,66,67,68,69].

## 3. Imaging Tools

Imaging tools without ionizing radiation should be employed during pregnancy. In this context, ultrasound (US) and magnetic resonance imaging (MRI) have several advantages over CT [30,61,68,70,71,72,73,74,75,76,77,78,79,80]. US is widely used, easy to perform, and has a low cost. However, the dependency operator reduces its diagnostic accuracy. MRIs have superior soft-tissue contrasts and functional applications compared to CT and US. However, the long examination time and high costs represent an obstacle, especially during an emergency. Abbreviated MRI protocols remove unnecessary sequences that negatively affect acquisition time. Although both methods are of proven utility, in some clinical settings, including emergencies, CT remains the method of problem solving and therefore of choice [27,50,51,52,53,60,73,77,81,82,83].

In any case, when a healthcare professional has to perform a diagnostic investigation, it is crucial that he keep in mind the fundamental principles of radiation protection, and this fact is even more important for a pregnant woman. Among these principles, the one that should be well known is the principle of optimization that the International Commission on Radiological Protection has named the “as low as reasonably achievable” (ALARA) principle, which states that, to obtain a diagnosis, the lowest possible dose level should be employed [10,13,24,25,26,27,28,29,30,31]. In addition, “the principle of justification” should be considered, i.e., the appropriateness of the imaging allows you to perform only necessary radiological procedures, avoiding unjustified exposure [10,13]. From a legal point of view, radiologists and obstetricians, before performing an imaging study, should have a signed informed consent form. Informed consent forms should be signed by all pregnant women for all diagnostic studies performed. Obtaining informed consent is good medical practice, since on the one hand it informs the patient about the possibility of alternative methods, albeit with different levels of accuracy, and, on the other hand, it makes known the risks and benefits of the methods used [14,24,32,34,54,75].

## 4. Clinical Setting: Abdominal Pain

The imaging tool choice to be employed in a pregnant patient’s abdominal assessment is complicated and should take into account the urgency in diagnosis, the main clinical hypotheses, the results of previous examinations, and the risks that these examinations have for the mother and fetus. The major non-obstetric clinical conditions that require an imaging assessment, because they are entities that can compromise the life of the mother and fetus, are those responsible for acute abdomen conditions, particularly appendicitis and biliary tract diseases, as well as an obstruction of the urinary tract, since it is responsible for pyelonephritis [9,30,33,34,35,36,37,38,39,40,41,42,43,44,45,46,47,48,49,50,51,52,53,54,55,56,57,58,59,60,61,62,63,64,65,66,67,68,84].

## 5. Acute Appendicitis

**Figure 1:**
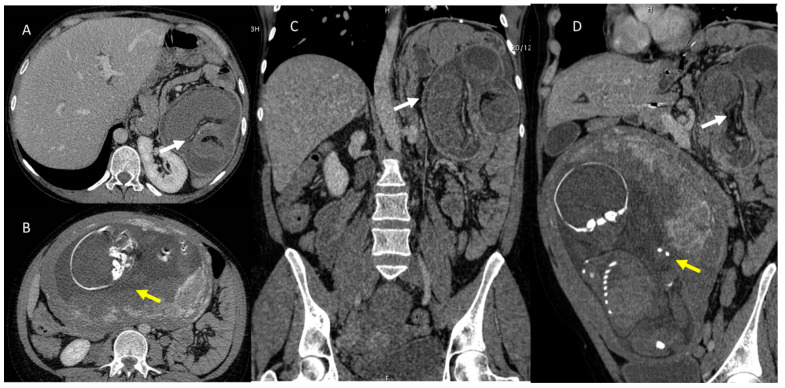
Pregnant woman (yellow arrows) with emergency room admission for abdominal pain, vomiting, and heart rhythm disturbances. US assessment reveals no specific findings. CT study in axial plane (**A**,**B**) and in coronal MPR plane (**C**,**D**) shows volvulus (white arrows) of the small bowel in the left epigastrium.

Acute appendicitis is the principal acute surgical abdomen condition in pregnancy, with an estimated prevalence of 50–70 cases/1000 patients [14,15,16]. This entity is correlated to a higher risk of premature childbirth and of perforation, as well as to higher rates of fetal morbidity [16]. In addition to the greater severity, the clinical symptoms may not help determine the diagnosis, since nausea, vomiting, and leukocytosis could be typical symptoms during pregnancy [16,59]. Moreover, several conditions can be responsible for abdominal pain, and a correct diagnosis is essential, hence the need for an imaging study. The choice of imaging modality should be tailored for the diagnosis of acute appendicitis in patients with a high degree of suspicion but should also allow diagnosis of other causes of abdominal pain to triage appropriate patient management. Issues related to the safety of the fetus during a diagnostic work-up is a major consideration. Abdominal US or MRI assessment is usually appropriate for the initial imaging of a pregnant woman with right lower quadrant pain with fever, leukocytosis, and suspected appendicitis. The reported US sensitivity in the diagnosis of acute appendicitis is variable, and the operator dependence could probably account for this variability in results [43]. Also, it should be considered that, in the terminal pregnancy phases, the appendix could be displaced by the gravid uterus, reducing the sensitivity of an US examination. On the other hand, the specificity is very high, at around 95% [14,16,43]. Therefore, US is the first imaging modality that should be employed both for diagnostic confirmation and for any differential diagnoses assessment (Figure 1). MRI and CT should be utilized as problem-solving tools [74]. Recent authors demonstrated that MRI has a higher accuracy compared to US [14,16], as well as to CT, with the advantages of greater availability and a shorter examination time than MRI, key factors during an emergency. However, due to the fetal risk linked to radiation exposure, it is to be considered a secondary instrument, with indications limited to cases that cannot be resolved with ultrasound or MRI [14,16,35,85].

Regarding the contrast medium, the ACR Committee on Drugs and Contrast Media has proposed the following concerning the performance of contrast-enhanced MRI examinations in pregnant patients: each case should be carefully considered by radiologists, and gadolinium-based contrast agent should be administered only when there is a potential benefit to the patient or fetus that outweighs the risk of fetal exposure to free gadolinium ions. In addition, abbreviated protocols including T2 half-Fourier acquisition single-shot turbo spin echo (HASTE) and diffusion-weighted imaging (DWI) sequences have been shown to reduce acquisition and interpretation times in an appendicitis diagnosis during an emergency with comparable accuracy compared to a full protocol [86].

Therefore, in this context, an abbreviated protocol probably remains an optimal choice.

## 6. Biliary Tract and Pancreatic Diseases

**Figure 2:**
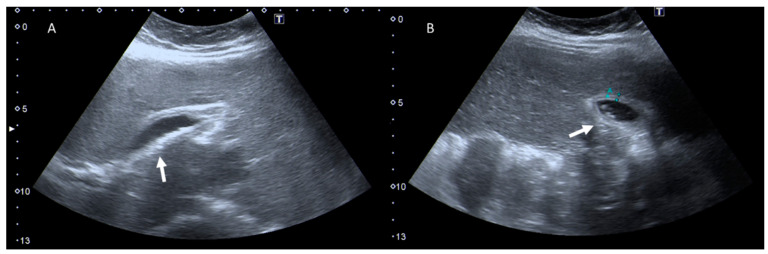
Pregnant woman with emergency room admission for right abdominal pain and fever. US assessment (**A**,**B**) shows (arrows) thickening of the gallbladder walls, with intraluminal hyperechoic material.

Acute cholecystitis is the second main cause of a non-obstetric surgical procedure in pregnancy (Figure 2), occurring in 1 of every 1600–10,000 cases. Other important conditions in this context are obstructive choledocholithiasis and biliary pancreatitis [14,17,67,87,88].

The incidence of acute pancreatitis (AP) in pregnancy varies and is approximately 1 in 1000 to 1 in 10,000 births. In the assessment of AP during pregnancy, four important issues should be resolved: (1) the AP diagnosis and ruling out of other causes; (2) the severity prediction; (3) the biliary etiology; and (4) the trimester of pregnancy. According to these issues and considering the latter, it is possible to define the diagnostic management.

Abdominal US is the initial imaging technique to identify a biliary etiology. Gallstones are detected by US in most cases. However, this tool has low sensitivity for the detection of common bile duct stones. For biliary tract diseases’ etiological clarification, MRI shows high sensitivity and specificity (98% and 84%, respectively). According to the Safety Committee of the Society for MRI [89], MRI should be employed during pregnancy if other non ionizing diagnostic tools studies are insufficient, or if this exam offers critical data that would otherwise require exposure to ionizing radiation.

Normally, in non-pregnant adults, CT is the most commonly used imaging tool in the diagnosis and assessment of the severity of AP. This tool is not suggested for pregnant patients due to the risk of radiation exposure to the fetus.

Therefore, US should be the first-line diagnostic test in cases of suspected acute biliary complications [14,16,90], while MRI should be a problem-solving tool for inconclusive US assessment and to assess the AP severity [35,88].

## 7. Ureterolithiasis

Urinary tract obstruction due to ureterolithiasis is a possible reason for abdominal pain in pregnancy. A proper diagnosis is required, since ureterolithiasis could cause pyelonephritis and sepsis, as well as a premature birth [18].

Although a pregnant woman could have physiological dilatation of the collecting system (hydroureteronephrosis), either due to extrinsic compression by the gravid uterus or due to hormonal changes that can cause ureteral relaxation, it is crucial to differentiate physiological hydronephrosis from an obstructive one, usually due to ureterolithiasis [14,16,18].

**Figure 3:**
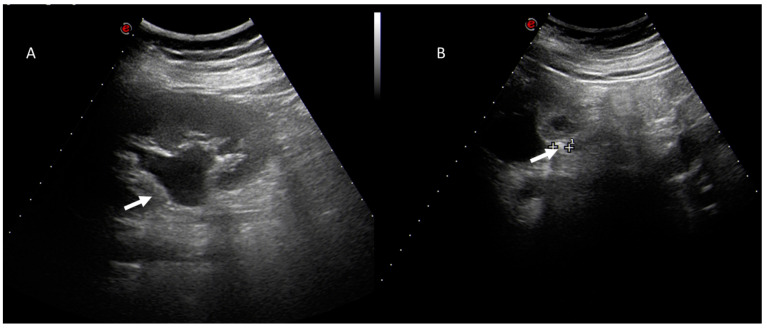
Pregnant woman with left flank pain. US assessment (**A**,**B**) shows hydroureteronephrosis (arrow) in (**A**) and distal ureteral calculi (arrow) in (**B**).

In the emergency diagnostic work-up of pregnant patients presenting with renal colic, the choice of diagnostic test should be tailored to the diagnosis, also considering other causes of flank pain and the safety to the fetus. Despite the relatively low sensitivity reported for identifying ureteral calculi (Figure 3) in pregnancy, US remains the first diagnostic tool that should be employed if there is suspicion of obstructive causes, since it is widely available, is low in cost, and allows for a hydroureteronephrosis assessment without ionizing radiation use [14,16,18,91]. Similarly to a pancreatitis diagnostic work-up, when US is inconclusive, the problem-solving method should be MRI [73,74,75,76]. In this pathological setting, MRI shows high accuracy in the diagnosis of urinary collection system obstruction as well as detecting renal complications. Contrast agents should be avoided according to the ACR Committee on Drugs and Contrast Media and abbreviated protocols, including T2-W, and pyelographic sequences could be a good alternative to the full protocol [16,18]. A CT study should be performed only if a definitive diagnosis of obstruction was not achieved with US and MRI and the patient’s clinical condition has deteriorated [29,84]. Particular attention must be paid to ensure the safety of the fetus, as it enters the field of view of an abdominopelvic CT scan, thus being exposed to the radiation beam. Although conventional protocols expose the fetus to a radiation dose estimated at 25 mGy, in this context, it would be preferable to employ low-dose protocols, using low tube current and voltage (160 mA and 140 kVp, respectively) to limit a fetal radiation dose to an estimated 11.7 mGy [10,16].

**Figure 4:**
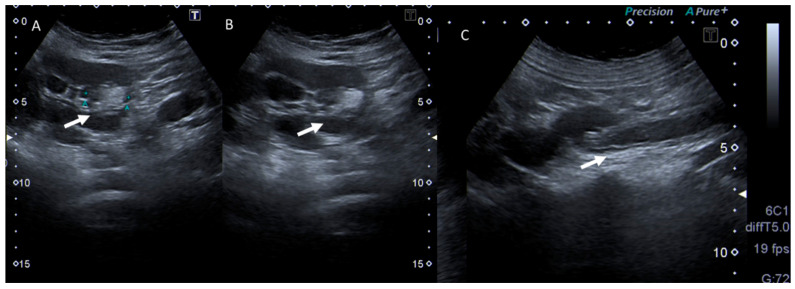
Pregnant woman with left flank pain and fever. US assessment shows in (**A**,**B**) hydroureteronephrosis with caliceal hyperechoic material (arrows) and thickening of the ureteral walls (**C**).

**Figure 5:**
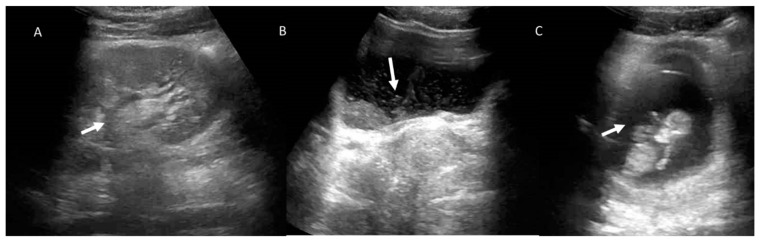
Pregnant woman (**C**) with abdominal pain and fever. US assessment shows hydroureteronephrosis with caliceal hyperechoic material (arrows) in (**A**) and thickening of the bladder walls, with intraluminal hyperechoic material in (**B**).

Acute pyelonephritis (APN) is a severe urinary infection (Figure 4) that can cause sepsis, shock, and death. Pregnancy increases the risk of complications from APN. Imaging studies are often employed to help with a diagnosis, identify causal features, and differentiate inferior infections (Figure 5) from renal involvement. The primary diagnostic tests used are CT, MRI, and US, although CT is usually not appropriate for a pregnant woman without other complications. An abdominal ultrasound is safe during pregnancy, is quick and portable, and does not require the use of contrast material. MRI should be used as a solution to the problem, which enables the detection of APN, scars, congenital anomalies of the kidneys, kidney abscesses, hydronephrosis, and pyonephrosis. According to the 2022 update of the ACR eligibility criteria for acute pyelonephritis [92], kidneys with color Doppler or MRI with or without contrast agents may be appropriate for imaging pregnant patients with no other complications.

## 8. Evaluation of the Pregnant Patient with Dyspnoea

The main causes of dyspnoea during pregnancy comprise pulmonary thromboembolism (PTE), pneumonia, asthma exacerbation, amniotic fluid aspiration, and pulmonary edema. Among them, the major cause of maternal mortality is PTE [58,65,93,94].

During pregnancy, the greatest risk of PTE is related to a state of hypercoagulability due to venous stasis and changes in coagulation factors; this condition results in a fivefold higher risk of deep vein thrombosis (DVT). Other predisposing features include advanced maternal age, thrombophilia, antiphospholipid syndrome, obesity, trauma, and surgery [19,23,51,64,95,96]. The clinical symptoms include dyspnoea, coughing, pleuritic chest pain, tachycardia, tachypnea, and hypoxemia [19,65,70,96]. Since PE is a leading cause of pregnancy-related mortality, the clinical suspicion of PTE must necessarily be confirmed by imaging [14,51,80,97,98].

The American Thoracic Society/Society of Thoracic Radiology (ATS/STR) Committee on Pulmonary Embolism in Pregnancy has released its clinical practice guideline [99], revised in seven endorsements, placing a high value on avoidance of work with radiation-associated testing, if possible [99]. In patients with signs of lower-extremity DVT and suspected PE, lower-extremity duplex US for assessment of DVT was suggested [99].

Although the choice between CT angiography (CTPA) and ventilation/perfusion scintigraphy (V/Q scan) in pregnant women remains the subject of debate [100,101,102], fetal radiation doses administered in the uterus during a properly performed examination do not pose an increased risk to the fetus. The ATS/STR statement recommends scintigraphy over CTA, mainly due to maternal, not fetal, radiation dose concerns [99]. In any case, since the diagnosis and treatment of PTE should not be delayed, the choice should be based primarily on which method is available, as well as on clinical judgment [19]. Scintigraphy has the advantage of lower maternal exposure, whereas CT allows for the detection of alternative diagnoses [17,20,64,97]. CT angiography is preferable and is also useful in cases where scan results are inconclusive and the level of clinical suspicion remains high [19,20,94,103,104,105,106].

According to the ACR Appropriateness Criteria for Suspected Pulmonary Embolism [107], in suspected PE pregnant patients with signs and symptoms of lower-extremity DVT, radiation tests should be avoided and lower-extremity duplex US should be employed for DVT assessment. The expert panels suggested no preferences regarding the CTPA or scintigraphy test. However, protocol study data are suggested. For CT scans, the administered dose of contrast agent should be reduced by a factor of two or more, with correspondingly longer acquisition times to obtain accurate imaging data. Regarding scintigraphy, if the perfusion test is normal, the ventilation test should be avoided [107].

## 9. Assessment of the Polytraumatized Pregnant Patient

Blunt abdominal trauma is the leading cause of traumatic injuries in pregnancy, with automobile accidents and falls being the most common etiologies. The evaluation of the polytraumatized pregnant patient is demanding, as the presence of the fetus implies the evaluation of two patients at risk [21,22], and therefore pregnant women should be managed in a medical center with the capacity to offer satisfactory care to both traumatized patients. Nonetheless, the mother’s survival remains the priority, and all treatment should be centered on her hemodynamic stability. In this scenario, all required diagnostic tests should be employed, since an inadequate diagnosis could cause maternal and fetal death [5]. So, it is clear that the benefits of imaging examinations outweigh any possible risk, even if using ionizing radiation is required [20,85].

**Figure 6:**
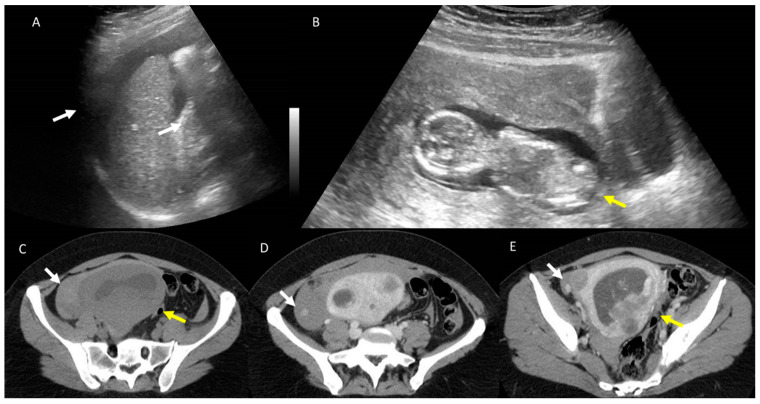
Polytraumatized (crash accident) pregnant woman (yellow arrows) admitted to emergency room. Fast US (**A** and **B**) shows abdominal free fluid. Unenhanced CT (**C**), white arrow shows intraabdominal blood with active bleeding (white arrows in **D**,**E**) after contrast medium administration.

Initial polytraumatized pregnant patient assessments (Figure 6) comprise radiography of the thorax/cervical spine, obstetric and abdominal US [50,108]. US is the first diagnostic tool since it is safe and is an integral part of the diagnostic management of all polytraumatized patients as a FAST examination. This approach allows for the evaluation of intraabdominal bleeding as free fluid and may also signal the presence of solid organ injury. Although FAST may be less sensitive in pregnant patients for abdominal injury, it remains highly specific. Equivocal findings require alternative techniques to identify traumatic injuries and to allow proper management [14,21], since no one necessary diagnostic study should be omitted [14,21]. Additional diagnostic assessments include the head, thorax, abdomen, and pelvis CT and, if appropriate, MRI for neurological injuries [72,109,110,111,112,113,114,115,116].

For blunt trauma, non-contrast CT should be avoided considering its lower sensitivity in detecting visceral and/or vascular lesions. Contrast agent administration is required to assess vascular and solid organ injury, and the protocol should include an angiography and a portal phase. Doubts remains on whole-body CT (WBCT), since no consensus exists for deciding which patients should receive WBCT versus selective CT studies.

Regarding abdominal and pelvic MRIs, there are a lack of supporting studies on MRI use in traumatized pregnant patients [117].

## 10. Neurological Complaints Assessment of Pregnant Patient

Pregnancy can be associated with several neurological illnesses such as headaches, preeclampsia, venous thrombosis, posterior reversible encephalopathy syndrome, subarachnoid hemorrhage, and pituitary diseases [22,56,66,102,110,113]. The diagnostic tools that could be employed include CT and MRI [102,106,114]. Although head CT does not include the fetus in the field of study and the fetus may only be exposed to a low radiation dose [22,56,81], MRI, which does not involve ionizing radiation, should nonetheless be preferred [71].

## 11. Discussion

Thousands of radiation workers and pregnant patients are exposed to ionizing radiation each year. Inadequate knowledge is the main cause of concern and of unnecessary pregnancy interruptions. However, even if for certain pregnant patients the dose does not increase fetus risk, the exposure is inappropriate in several settings, causing the fetus an unjustified increased risk [11,79,81,104,108]. In this context, it is critical that healthcare professionals keep in mind the fundamental principles of radiation protection and utilize the proper diagnostic tool according to the clinical setting (Table 1). In fact, US and MRI should be preferred during pregnancy. However, in a critical setting, such as trauma, when the benefits outweigh the potential risks to the fetus, CT should be performed, although the study protocol should be optimized [21,55,57,78,98,112,113,114,115,116,117,118,119,120,121,122,123]. In addition, examination in anatomical regions far from the fetus, such as those on the chest, skull, or extremities, can typically be performed safely at any stage during pregnancy, with adequate bundle collimation [10,39]. When the uterus is directly exposed to the bundle, there may be high exposure to the fetus with absorbed doses that may reach or exceed 50 mGy [44]. In this case, care should be taken to minimize the dose absorbed by the fetus and an estimate of the dose to which the fetus will be exposed should be assessed [29,37,45,46,51,52,53,54,55,56,57,58,59,60,61,62,63,100]. This may not be possible in an emergency setting.

Regarding patient gonadal and fetal shielding during X-ray-based diagnostic imaging, it should be discontinued as a routine practice. Patient shielding may jeopardize the benefits of undergoing radiological imaging. Use of these shields during X-ray-based diagnostic imaging may obscure anatomical information or interfere with the automatic exposure control of the imaging system. These effects can compromise the diagnostic efficacy of the exam, or actually result in an increase in the patient’s radiation dose. Because of these risks and the minimal to nonexistent benefit associated with fetal and gonadal shielding, the American Association of Physicists in Medicine (AAPM) recommends that the use of such shielding be discontinued [119].

All procedures with fluoroscopic guidance or CT should be optimized to achieve the clinical target with the minimum radiological exposure required, taking into account the resources and technologies available [28,101]. Several strategies for CT protocol optimization exist, such as tube modulation, *z*-axis overscan, scan length limitation, kV modulation in smaller size and younger patients, use of iterative reconstruction algorithms, and use of machine learning and artificial intelligence algorithms [10,11,23,52,74,75,76,90,91,92,93,105,111,113,114,115]. Dose optimization to the patient and the fetus does not result in the delivery of the minimum dose [38,62,63,64,65,66,111], and it is critical to achieve maximum dose reduction, consistent with acceptable image quality [3,58,79,83,101]. There are simple technical measures that make it possible to achieve this purpose, such as fetus exclusion from the primary beam trajectory, the use of collimation modules, as well as the appropriate selection of the many technical factors affecting the dose [40,41,49,50,51,52,53,54,55,56]. General guidelines for managing the radiological dose to the patient are important for the optimization of pregnant doses and related conception products [42,69,95,100,105,110,115,118].

## 12. Conclusions

Optimization, regardless of the type of clinical question, always remains the only effective solution for the correct management of the diagnosis, especially in the case of pregnant patients. It is critical that healthcare professionals keep in mind the fundamental principles of radiation protection and utilize the proper diagnostic tool according to the clinical setting. Although US and MRI should be preferred during pregnancy, in a critical setting, such as trauma, when the benefits outweigh the potential risks to the fetus, CT should nevertheless be preferred. All CT procedures should be optimized to achieve the clinical target with the minimum radiological exposure required. Several strategies for CT protocol optimization exist, such as tube modulation, *z*-axis overscan, scan length limitation, kV modulation in smaller size and younger patients, use of iterative reconstruction algorithms, and use of machine learning and artificial intelligence algorithms.

## Figures and Tables

**Table 1:** Clinical setting and imaging tools utilized.

Clinical Setting	Primary Assessment	Secondary Assessment
Acute appendicitis	US	MRI and CT
Biliary tract and pancreatic diseases	US	MRI CT with contrast medium for acute pancreatitis severity assessment
Ureterolithiasis Acute pyelonephritis	US	MRI CT study without contrast agent should be performed only if a definitive diagnosis of obstruction was not achieved with US and MRI and the patient’s clinical condition has deteriorated. CT with contrast agent for Acute pyelonephritis assessment.
Pulmonary thromboembolism	Lower-extremity duplex US for DVT assessment	CT angiography
Polytraumatized patient	FAST US	Head, thorax, abdomen, and pelvis CT with contrast medium. MRI for neurological injuries
Neurological Diseases	MRI	

## Data Availability

All data are reported in the manuscript.
